# Associations of IFT20 and GM130 protein expressions with clinicopathological features and survival of patients with lung adenocarcinoma

**DOI:** 10.1186/s12885-022-09905-6

**Published:** 2022-07-22

**Authors:** Lianfeng Li, Yaobing Chen, Wei Liao, Qimei Yu, Hui Lin, Yuqin Shi, Ling Zhang, Guoqing Fu, Zhenyu Wang, Xi Li, Xianrong Kong, Ting Zhou, Lingzhi Qin

**Affiliations:** 1 Department of Occupational and Environmental Health, School of Public Health, Wuhan University of Science and Technology, Wuhan, 430065 Hubei China; 2 Hubei Province Key Laboratory of Occupational Hazard Identification and Control, Wuhan University of Science and Technology, Wuhan, 430065 Hubei China; 3 Institute of Pathology, Tongji Hospital, Tongji Medical College, Huazhong University of Science and Technology, Wuhan, 430030 Hubei China; 4 Department of Respiratory and Critical Care Medicine, Fifth Hospital in Wuhan, Wuhan, 430050 Hubei China; 5 Department of Basic Medicine, Medical College, Wuhan University of Science and Technology, Wuhan, 430065 Hubei China; 6 Department of Radiology, Tongji Hospital, Tongji Medical College, Huazhong University of Science and Technology, Wuhan, 430030 Hubei China

**Keywords:** Lung adenocarcinoma, IFT20, GM130, Clinicopathological features, Overall survival

## Abstract

**Background:**

Lung cancer is the leading cause of malignancy-related mortality and lung adenocarcinoma accounts for about 40% of lung malignancies. The aim of this study was to investigate the associations of intraflagellar transport protein 20 (IFT20) and Golgi matrix protein 130 (GM130) expression with clinicopathological features and survival in patients with lung adenocarcinoma.

**Methods:**

The expressions of IFT20 and GM130 protein in cancerous and matched adjacent lung tissues of 235 patients with lung adenocarcinoma were assessed by tissue microarray and immunohistochemistry, which were indicated by the mean optical density (IOD/area), the rate of positive staining cells and staining intensity score. The correlation between IFT20 and GM130 protein was assessed by Spearman’s rank correlation. Associations of IFT20 and GM130 protein expression with clinicopathological features of patients were analyzed by multivariate logistic regression models. The survival analysis of patients was performed by Cox proportional hazard regression models.

**Results:**

With adjustment for multiple potential confounders, each one-point increase in IFT20 protein staining intensity score was significantly associated with 32% and 29% reduced risk for TNM stage in II ~ IV and lymphatic metastasis of patients, respectively (*P* < 0.05). And each one-point increase in GM130 protein staining intensity score was associated with a significant reduction in the risk of poor differentiation and tumors size > 7 cm by 29% and 38% for lung adenocarcinoma patients, respectively (*P* < 0.05). In stratified Cox model analysis, enhanced IFT20 staining intensity score was significantly decreased the risk of death by 16% for patients without distant metastasis. And elevated the IOD/area of GM130 expression significantly decreased the death risk of lung adenocarcinoma patients with tumor size > 7 cm or distant metastasis by 54% and 65%, respectively (*P* < 0.05).

**Conclusion:**

IFT20 and GM130 protein expressions were negatively associated with tumor differentiated types, size, TNM stage and lymphatic metastasis of lung adenocarcinoma. Both IFT20 and GM130 proteins have some protective effects on the survival of lung adenocarcinoma patients with specific clinicopathological features.

**Supplementary Information:**

The online version contains supplementary material available at 10.1186/s12885-022-09905-6.

## Introduction

Lung cancer is the leading cause of malignancy-related mortality, resulting in more deaths than breast, prostate, colorectal and brain cancers [1]. Lung adenocarcinoma is the most common histological subtype in non-small cell lung cancer (NSCLC), which accounts for about 40% of lung malignancies [2]. The average 5-year survival rate of lung adenocarcinoma is only 18%, mainly due to invasion and metastasis of tumor cells, diagnosed at advanced stages or lack of effective treatment strategies [3]. The tumor invasion and metastasis have a significant impact on the quality of life and overall survival (OS) of patients with lung adenocarcinoma [4]. Thus, early detection of tumor invasion and metastasis is crucial to improve the survival rate of patients with lung adenocarcinoma.

Primary cilia are antenna-like organelles protruding from the surface of most eukaryotic cells that participate in cell proliferation, differentiation and migration [5]. Some studies have reported that the number of primary cilia markedly decreased in the transformation of many tumor cells, including breast, pancreas, ovarian, and kidney cancer cells, whereas it was significantly higher in cancerous tissues of lung adenocarcinoma, colon adenocarcinoma and follicular lymphoma than that in normal control tissues [6]. Primary cilia require a series of intraflagellar transport (IFT) protein complexes that participate in the transportation of ciliary signaling proteins as well as the process of cellular protrusion and resorption to assemble and maintain their structures [7]. IFT20, the smallest IFT protein, localizes at the cis-Golgi apparatus that regulates the assembly and maintenance of primary cilia by sorting of ciliary cargo from the cis-Golgi to the base of the cilium [8, 9]. Down-regulated expression of IFT20 mRNA or protein could not only inhibit ciliary assembly and decrease the quantity of primary cilia in mammalian cells, but also reduce invasion and metastasis of colorectal cancer and osteosarcoma cells [8–10]. A new study found that IFT20 promotes collective invasion of colorectal cancer by regulating organization of Golgi-associated, stabilized microtubules and Golgi polarity in colorectal cancer cells at the invasive front [9].

Recent studies indicated that tumor’s invasion and metastasis often require cell polarization and migration [10], which need the Golgi to adopt a ribbon-like morphology in promoting polarized secretion to the cell surface [11, 12]. As a cis-Golgi matrix protein, GM130 is essential for the maintenance of Golgi structure and protein transportation that involves in cell polarization and migration [13, 14]. Expression of GM130 mRNA or protein was significantly lower in tissues of colorectal adenocarcinoma and breast cancer than that in matched normal tissues, and the depletion of GM130 mRNA enhanced the invasiveness of breast cancer cells [15, 16]. Remarkably, IFT20 protein localized at Golgi could combine with GM130 and A-kinase anchor protein 450 (GM130-AKAP450) complex to regulate cellular microtubules nucleation, which contributes to Golgi ribbon formation in achieving polarized secretion for cell migration and invasion [10, 13]. Thus, both IFT20 and GM130 proteins might play important roles in the invasion and metastasis of various malignant tumors, but their roles in the tumorigenesis and development of lung adenocarcinoma remains unclear so far.

Hence, we determined the expressions of IFT20 and GM130 protein in cancerous and matched adjacent lung tissues of patients with lung adenocarcinoma by tissue microarray and immunohistochemistry to investigate their potential roles in the development of lung adenocarcinoma and the relationships between their expressions and survival of patients with lung adenocarcinoma.

## Materials and methods

### Study population

A total of 235 patients with lung adenocarcinoma were selected from one hospital in Wuhan city in China from March 2010 to September 2015 by simple random sampling, and followed up to June 2018. All the patients underwent lung cancer surgery and were finally diagnosed with lung adenocarcinoma by pathological biopsy. This study was approved by the Ethnics and Human Subject Committees of Tongji hospital at Huazhong University of Science and Technology. All participants enrolled in this study signed written informed consent for participation, storage and use of surgically-removed cancerous and matched adjacent tissues. All methods of this study were carried out in accordance with relevant guidelines and regulations.

### Data and tissue samples collection

We collected basic information such as age, gender, smoking status, smoking amount, and clinicopathological features including differentiated types of tumor cells, tumor size, tumor node metastasis (TNM) stage, lymphatic or distant metastasis, cell proliferation index (Ki67), chemotherapy, and radiotherapy. Smoking amount (pack-years) for each smoker was calculated as packs of cigarettes per day multiplied by years of smoking. The differentiated types of lung adenocarcinoma were classified into micropapillary and solid, acinar and papillary, and lepidic types, which are identified as poorly, moderately and well differentiated tumors according to the criteria established by World Health Organization [17]. The TNM stage of patients with lung adenocarcinoma was diagnosed based on the 7th edition (2009) of the American Joint Committee on Cancer for Lung Cancer [18]. And the cancerous and matched adjacent lung tissues of each patient were collected for histological analysis.

### Tissue chips preparation

Small pieces of tissue samples were fixed in 4% paraformaldehyde for 24 h, and then dehydrated, paraffin-embedded, sliced and stained with hematoxylin and eosin (HE), which were examined by microscope to determine the sampling location of the chip. After heating the paraffin block, the target samples taken out by a sampler were inserted into the prepared paraffin block receptor hole in sequence. The samples were merged at 60 ~ 65 °C for 20 min using tissue chip fusion instrument (BP0100, Biossci Company). The melted block was then put into a wax mold, embedded in paraffin and cut into 4 μm thick sections.

### Immunohistochemistry (IHC)

The sections were deparaffinized, rehydrated, rinsed with distilled water, and repaired at high temperature and pressure for 1 ~ 2 min. After cooled to room temperature and washed with Tris-buffer solution (TBS) three times, the sections were incubated with fresh 3% hydrogen peroxide at room temperature for 20 min and blocked with 10% normal goat serum for 20 min. The sections were then incubated with primary antibodies (IFT20, 1:200, proteintech, 13,615–1-AP; GM130, 1:200, abcam, ab52649) overnight at 4 °C. After incubated at room temperature for 15 min, the sections were washed by TBS three times and then incubated with secondary antibodies (50 μl DAKO) for another 25 min at room temperature. Then the sections were washed three times, stained with diaminobenzidine and counterstained by hematoxylin.

The expressions of positive staining IFT20 and GM130 protein were indicated by the mean optical density (IOD/area), the rate and intensity of positive staining cells. The average IOD/area and the rate of positively staining cells in five representative views (original objective 400×) from each section were analyzed by Image J software version 1.2.4 (the National Institutes of Health, NIH free software, Bethesda, MD, USA). The average IOD/area of these views represented the relative expressions of positive staining IFT20 and GM130 protein, and the rate of positive stained cells was equal to positive cells number/total cells number× 100%. The staining intensity of positive cells of each section was denoted by staining intensity score (0 = no staining; 1 = weak staining; 2 = intermediate staining; 3 = strong staining), which was independently evaluated by five professionally trained persons without knowledge of the clinicopathological data of patients. The final score was determined as the consistent results made by three or more persons. D value is equal to the difference value of protein expression between cancerous tissue and matched adjacent tissue. The patients were divided into negative and positive groups based on the D values of IFT20 or GM130 protein staining intensity score, D value ≤0 was defined as the negative group, whereas D value > 0 was regarded as the positive group.

### Statistical analysis

Continuous variables were divided into normal and non-normal distribution, which were compared by Student’s t test and Wilcoxon rank sum test, respectively. Categorical variables were analyzed by chi-square test or Fisher’s exact test. The correlation between IFT20 and GM130 protein was assessed by Spearman’s rank correlation. Associations of IFT20 and GM130 protein expressions with clinicopathological features of lung adenocarcinoma patients were assessed by multivariate logistic regression models, adjusting for multiple potential confounders including age, gender, smoking status, smoking amount. The survival analysis of patients with lung adenocarcinoma after lung cancer surgery was performed by Cox proportional hazard regression models. All statistical analyses were carried out with SPSS version 26.0 (SPSS Inc., Chicago, IL), and *P* < 0.05 was considered statistically significant.

## Results

### The basic characteristics of all participants

As all the patients were divided into two groups based on the negative and positive expressions of IFT20 and GM130 protein, the number and proportion of IFT20 and GM130 positive expression patients were 168 (71.5%) and 151 (64.3%), respectively, In Table 1, we found that the amount of smoking was significantly less in IFT20 and GM130 positive expression groups than that in the negative groups (*P* < 0.05). Compared with IFT20 negative group, the proportion of male, current smoking patients was lower in IFT20 positive expression group, which was statistically significant (*P* < 0.05). The percent of clinicopathological features such as T3 ~ T4 stages, N2 ~ N3 stages and lymphatic metastasis was also much lower whereas the average OS of patients was significantly longer in IFT20 positive expression group than those in IFT20 negative expression group (*P* < 0.05). Besides, the proportion of poorly differentiated type cells was significantly lower in GM130 positive expression group when compared with the negative expression group (*P* < 0.05). And the average size of tumor (4.32 ± 1.89 cm) in GM130 positive expression group is also considerably smaller than that (5.02 ± 2.67 cm) in the negative expression group *(P* < 0.05).

**Table 1:** The characteristics of all patients with lung adenocarcinoma

Variables	IFT20 protein expression		*P* value	GM130 protein expression		*P* value
	Negative (*n* = 67)	Positive (*n* = 168)		Negative (*n* = 84)	Positive (*n* = 151)	
Age (years, Mean ± SD)	57.25 ± 9.58	56.20 ± 9.86	0.458	57.36 ± 9.62	56.03 ± 9.86	0.318
Gender, (Male, n, %)	47(70.15)	90(53.57)	0.024	55(65.48)	82(54.30)	0.096
Smoking status (n, %)			0.009			0.177
Current smoking	28(41.79)	44(26.19)		27(32.14)	45(29.80)	
Never smoking	28(41.79)	107(63.69)		43(51.19)	92(60.93)	
Former smoking	11(16.42)	17(10.12)		14(16.67)	14(9.27)	
Smoking amount, pack-year	22.05 ± 25.24	13.30 ± 23.06	0.011	20.67 ± 27.98	13.09 ± 21.04	0.020
Differentiated types of tumors (n, %)			0.377			0.049
Poorly	20(29.85)	51(30.36)		33(39.29)	38(25.17)	
Moderately	39(58.21)	85(50.60)		36(42.86)	88(58.28)	
Well	8(11.94)	32(19.05)		15(17.86)	25(16.56)	
Ki67			0.441			0.222
< 50%	56(83.58)	133(79.17)		64(76.19)	125(82.78)	
≥50%	11(16.42)	35(20.83)		20(23.81)	26(17.22)	
Tumor size (cm)	4.93 ± 2.56	4.43 ± 2.06	0.117	5.02 ± 2.67	4.32 ± 1.89	0.019
T stages (n, %)			0.012			0.206
T1 ~ T2	36(53.73)	119(70.83)		51(60.71)	104(68.87)	
T3 ~ T4	31(46.27)	49(29.17)		33(39.29)	47(31.13)	
N stages (n, %)			0.048			0.084
N0 ~ N1	19(28.36)	71(42.26)		26(30.95)	64(42.38)	
N2 ~ N3	48(71.64)	97(57.74)		58(69.05)	87(57.62)	
M stages (n, %)			0.193			0.785
M0	50(74.63)	138(82.14)		68(80.95)	120(79.47)	
M1	17(25.37)	30(17.86)		16(19.05)	31(20.53)	
TNM stages (n, %)			0.182			0.613
I stage	9(13.43)	43(25.60)		15(17.86)	37(24.50)	
II stage	12(17.91)	25(14.88)		15(17.86)	22(14.57)	
III stage	29(43.28)	70(41.67)		38(45.24)	61(40.40)	
IV stage	17(25.37)	30(17.86)		16(19.05)	31(20.53)	
Chemotherapy/radiotherapy (yes, n, %)	39(58.21)	110(65.48)	0.296	51(60.71)	98(64.90)	0.523
Lymphatic metastasis (yes, n, %)	48(71.64)	97(57.74)	0.048	58(69.05)	87(57.62)	0.084
Distant metastasis (yes, n, %)	17(25.37)	30(17.86)	0.193	16(19.05)	31(20.53)	0.785
Alive (yes, n, %)	14(20.90)	42(25.00)	0.505	15(17.86)	41(27.15)	0.109
Overall survival (years, Mean ± SD)	2.42 ± 1.64	3.00 ± 1.71	0.018	2.57 ± 1.73	2.99 ± 1.69	0.066

### Expressions of IFT20 and GM130 protein in lung tissues of patients with lung adenocarcinoma

**Fig. 1:**
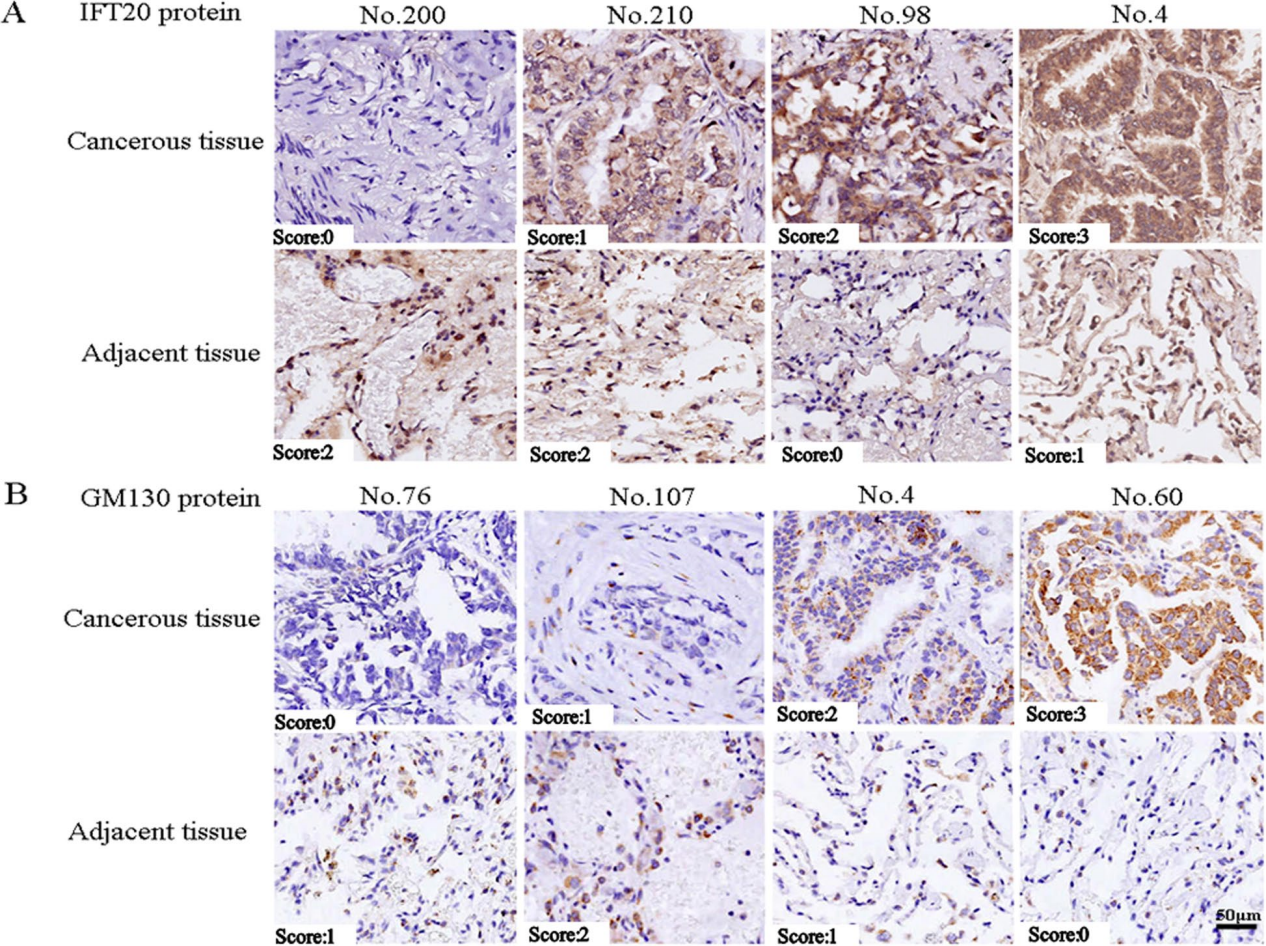
Expressions of IFT20 and GM130 protein in cancerous and matched adjacent tissues of patients with lung adenocarcinoma (*tissue chip, IHC, objective × 400*). A) IFT20 protein. B) GM130 protein

As shown in Fig. 1 A and B, the expressions of IFT20 and GM130 protein were both positively expressed in cancerous and matched adjacent lung tissues of patients with lung adenocarcinoma. By semi-quantitative analysis, IFT20 expression (IOD/area, rate of positive staining cells and staining intensity score) and GM130 protein staining intensity score was significantly higher in cancerous tissues than those of matched adjacent tissues (*P* < 0.001) (Table 2). Additionally, we found that the IOD/Area and staining intensity score of IFT20 protein were positively correlated with GM130 protein corresponding expression in cancerous tissues (*r =* 0.223 and *r =* 0.492, *P* < 0.001) and adjacent tissues (*r =* 0.385 and *r =* 0.424, *P* < 0.001) (Supplemental Table 1).

**Table 2:** Expressions of IFT20 and GM130 protein in cancerous and matched adjacent tissues of patients with lung adenocarcinoma by semi-quantitative analysis

Proteins	Variables	Cancerous tissue	Adjacent tissue	D value
IFT20	IOD/area	0.201 (0.175, 0.230)*	0.177 (0.158, 0.198)	0.021 (−0.006, 0.052)
	Rate of positive cells	31.05 (20.14, 42.09)*	17.10 (12.01, 24.42)	12.47 (3.10, 22.83)
	Staining intensity score	3.0 (2.0, 3.0)*	1.0 (1.0, 2.0)	1.0 (0.0, 2.0)
GM130	IOD/area	0.095 (0.079, 0.109)	0.110 (0.095, 0.123)	−0.014 (− 0.031, 0.005)
	Rate of positive cells	9.37 (3.03, 19.00)	10.21 (6.29,1 3.61)	−0.16 (−6.36, 7.68)
	Staining intensity score	2.0 (1.0, 2.0)*	1.0 (0.0, 1.0)	1.0 (0.0, 2.0)

*P < 0.05, comparison between cancerous and adjacent tissues

### Associations of IFT20 and GM130 protein expressions with clinicopathological features of patients with lung adenocarcinoma

To illustrate the relationships of IFT20 and GM130 protein expressions with clinicopathological features, multivariate logistic regression models were analyzed by adjusting for multiple potential confounders such as age, gender, smoking status, and smoking amounts. As shown in Table 3, each one-point increase in the D value of IFT20 protein staining intensity score was significantly associated with a 32% and 29% reduced risk for TNM stage in II ~ IV and lymphatic metastasis of patients, respectively (*P* < 0.05). The adjusted odd ratio (OR) and 95% confidence interval (CI) for rate of IFT20 positive cells in the highest quartile was 0.44 (0.20, 0.93) in comparison with that in the lowest quartile for patients with lymphatic metastasis. Although there was no association between IFT20 protein with types of tumor cell differentiation, tumor size or distant metastasis, each one-point increase in the D value of GM130 staining intensity score significantly reduced the risk of poorly differentiated type and tumors size> 7 cm by 29% and 38%, respectively (*P* < 0.05). In categorical analysis, there was also statistically negative relationship of GM130 protein expression (IOD/area or rate of positive cells) with the risk of poorly differentiated type cells, tumor size > 7 cm or TNM stage in II ~ IV. Furthermore, elevated rate of GM130 positive cells was also significantly associated with decreased risk of poorly differentiated type cells in a dose-dependent manner (*P*_trend_ < 0.05). However, no significant relationships were observed between GM130 expression (IOD/area, rate of positive cells or staining intensity score) and clinicopathological features of lymphatic or distant metastasis (*P* > 0.05).

**Table 3:** Associations of IFT20 and GM130 protein expressions with clinicopathological features of patients with lung adenocarcinoma

Proteins	D Value	Differentiated types		Tumor size		TNM stage		Lymphatic metastasis		Distant metastasis	
		Poorly/moderately to well	OR(95%CI)	> 7 cm/≤7 cm	OR(95%CI)	II ~ IV/I	OR(95%CI)	Yes/No	OR(95%CI)	Yes/No	OR(95%CI)
IFT20	IOD/area	71/164	0.96 (0.72, 1.28)	25/210	0.71 (0.46, 1.09)	183/52	0.84 (0.62, 1.16)	145/90	0.82 (0.63, 1.08)	47/188	1.01 (0.72, 1.41)
	Q1(<−0.006)	20/38	reference	8/50	reference	49/9	reference	37/21	reference	13/45	reference
	Q2(−0.006 ~ 0.0207)	14/45	0.57 (0.25, 1.29)	8/51	0.92 (0.30, 2.82)	49/10	0.82 (0.31, 2.23)	40/19	1.13 (0.52, 2.45)	12/47	0.82 (0.33, 2.05)
	Q3(0.0207 ~ 0.052)	18/42	0.85 (0.39, 1.87)	5/55	0.60 (0.17, 2.05)	42/18	0.44 (0.17, 1.04)	36/24	0.81 (0.38, 1.72)	9/51	0.62 (0.24, 1.63)
	Q4(> 0.052)	19/39	0.97 (0.44, 2.12)	4/54	0.39 (0.10, 1.47)	43/15	0.50 (0.20, 1.28)	32/26	0.65 (0.30, 1.38)	13/45	0.95 (0.38, 2.34)
	*P*_trend_	0.922		0.131		0.080		0.186		0.823	
	Rate of positive cells	71/164	1.00 (0.98, 1.01)	25/210	1.00 (0.97, 1.02)	183/52	0.99 (0.97, 1.01)	145/90	0.99 (0.97, 1.01)	47/188	0.99 (0.97, 1.01)
	Q1(< 3.096)	17/41	reference	5/53	reference	49/9	reference	40/18	reference	15/43	reference
	Q2(3.096 ~ 12.466)	20/40	1.27 (0.58, 2.79)	6/54	1.17 (0.32, 4.27)	46/14	0.58 (0.23, 1.48)	35/25	0.63 (0.30, 1.34)	11/49	0.68 (0.27, 1.68)
	Q3(12.466 ~ 22.828)	21/37	1.41 (0.64, 3.10)	10/48	2.39 (0.73, 7.86)	47/12	0.67 (0.26, 1.77)	41/17	1.09 (0.49, 2.40)	10/48	0.57 (0.23, 1.43)
	Q4(> 22.828)	13/46	0.72 (0.31, 1.67)	4/55	0.76 (0.18, 3.16)	41/17	0.40 (0.16, 1.01)	29/30	0.44 (0.20, 0.93)	11/48	0.61 (0.24, 1.51)
	*P*_trend_	0.414		0.912		0.086		0.07		0.331	
	Staining intensity score	71/164	0.99 (0.74, 1.34)	25/210	0.66 (0.44, 1.01)	183/52	0.68 (0.47, 0.96)	145/90	0.71 (0.53, 0.94)	47/188	0.82 (0.59, 1.14)
GM130	IOD/area	71/164	0.97 (0.73, 1.29)	25/210	0.82 (0.56, 1.20)	183/52	1.10 (0.82, 1.48)	145/90	0.97 (0.74, 1.27)	47/188	1.13 (0.78, 1.63)
	Q1(<−0.031)	23/36	reference	11/48	reference	48/11	reference	39/20	reference	7/52	reference
	Q2(−0.031 ~ − 0.014)	15/45	0.52 (0.24, 1.14)	3/57	0.14 (0.03, 0.60)	46/14	0.67 (0.27, 1.66)	38/22	0.81 (0.37, 1.74)	13/47	1.88 (0.67, 5.29)
	Q3(−0.014 ~ 0.005)	20/37	0.85 (0.40, 1.80)	3/54	0.23 (0.06, 0.92)	43/14	0.75 (0.30, 1.88)	35/22	0.82 (0.38, 1.77)	12/45	2.10 (0.74, 5.92)
	Q4(> 0.005)	13/46	0.44 (0.20, 0.99)	8/51	0.44 (0.15, 1.33)	46/13	0.70 (0.28, 1.78)	33/26	0.57 (0.27, 1.23)	15/44	2.06 (0.75, 5.66)
	*P*_trend_	0.102		0.204		0.508		0.226		0.17	
	Rate of positive cells	71/164	0.97 (0.95,0.99)	25/210	0.97 (0.94, 1.01)	183/52	1.00 (0.98, 1.03)	145/90	0.99 (0.98, 1.02)	47/188	1.00 (0.98, 1.03)
	Q1(<−6.377)	23/36	reference	10/49	reference	48/10	reference	38/21	reference	12/47	reference
	Q2(−6.377 ~ −0.164)	22/36	1.01 (0.48, 2.15)	4/54	0.28 (0.08, 1.03)	48/12	0.78 (0.31, 1.99)	40/18	1.23 (0.57, 2.65)	13/45	1.05 (0.42, 2.63)
	Q3(−0.164 ~ 7.683)	13/47	0.42 (0.19, 0.95)	5/55	0.33 (0.10, 1.08)	39/20	0.38 (0.16, 0.92)	29/31	0.52 (0.25, 1.08)	9/51	0.71 (0.26, 1.88)
	Q4(> 7.683)	13/45	0.48 (0.21, 1.09)	6/52	0.45 (0.14, 1.45)	48/10	0.95 (0.36, 2.51)	38/20	1.05 (0.49, 2.25)	13/45	1.02 (0.41, 2.54)
	*P*_trend_	0.024		0.294		0.897		0.073		0.948	
	Staining intensity score	71/164	0.71 (0.55, 0.91)	25/210	0.62 (0.41, 0.93)	183/52	0.95 (0.72, 1.26)	145/90	0.79 (0.62, 1.01)	47/188	1.03 (0.77, 1.38)

Estimates were adjusted for age, gender, smoking status, and smoking amounts.

### Influence of IFT20 and GM130 protein expressions on the survival of patients with lung adenocarcinoma

**Fig. 2:**
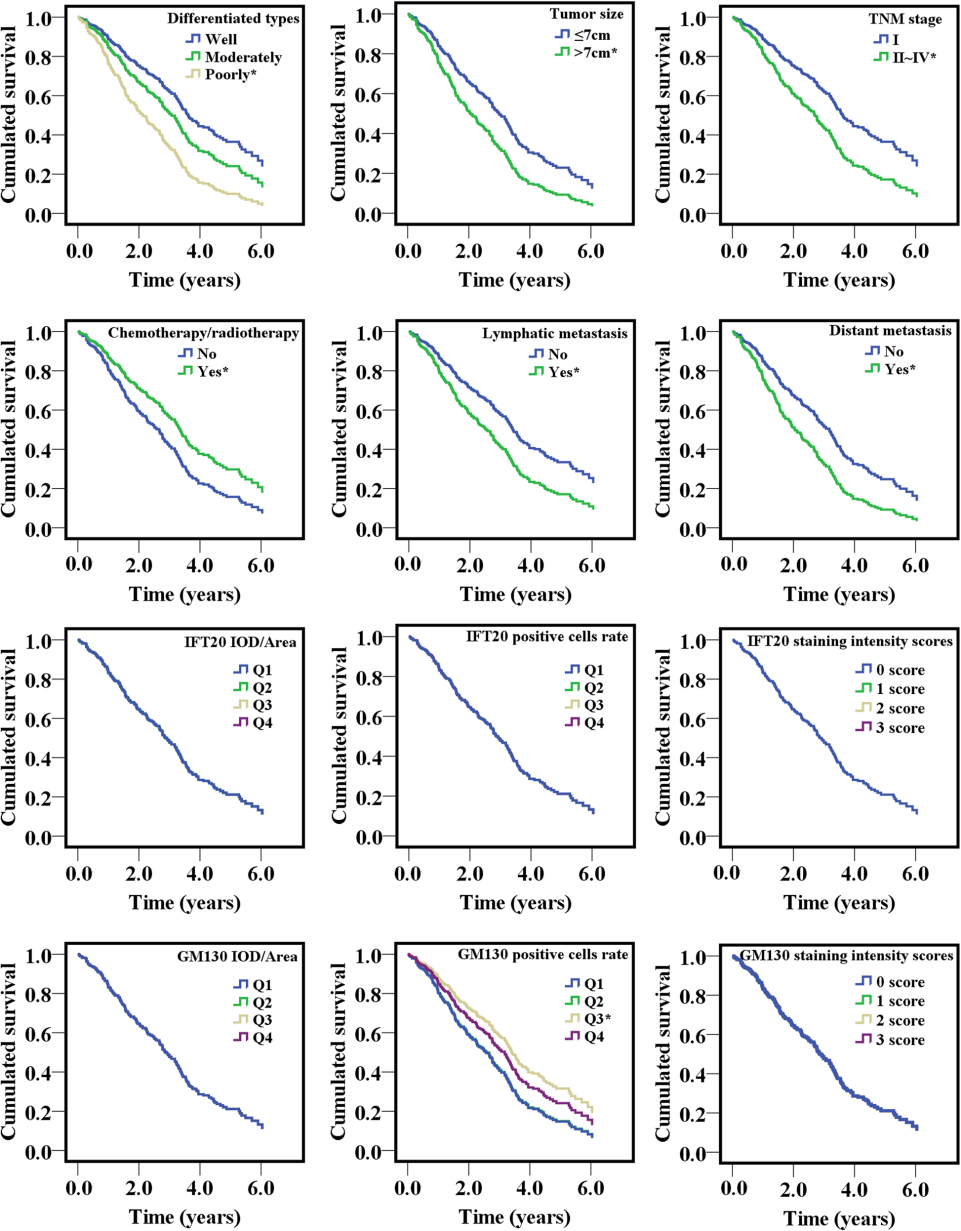
The survival curves of clinicopathological features and IFT20 and GM130 protein expressions for patients with lung adenocarcinoma. *P < 0.05, compared with the blue line which is defined as reference line. The only one blue curve means that the survival curves for the Q2, Q3 and Q4 levels of IFT20 and GM130 protein overlapped with the reference line, respectively, after adjusting potential confouders such as gender, age, smoking status and smoking amount

**Fig. 3:**
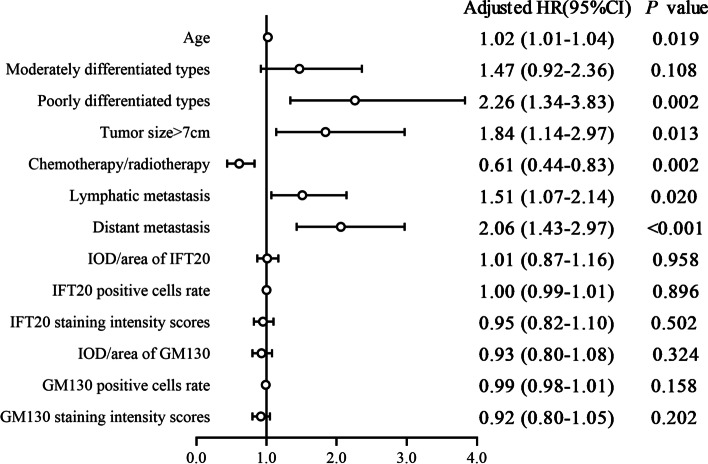
The forest plot of Cox proportional hazard regression models for patients with lung adenocarcinoma. To exclude collinearity, the three types' expressions of IFT20 and GM130 protein (IOD/area, rate of positive cells, and staining intensity score) were separately included in the model

Cox proportional hazard regression models were conducted to evaluate the influence of IFT20 and GM130 protein expressions on the survival of lung adenocarcinoma patients after surgery. As shown in Fig. 2, the expressions of IFT20 and GM130 protein had no significant effect on the survival of patients. However, the OS of patients was significantly reduced by poorly differentiated type cells, tumor size > 7 cm, TNM stage II ~ IV, lymphatic and distant metastasis, whereas it was prolonged by chemotherapy and radiotherapy (*P* < 0.05). Furthermore, the clinicopathological features of poorly differentiated type, tumor size > 7 cm, TNM stage II ~ IV, lymphatic and distant metastasis had significantly positive influences on the increased risk of death, with the hazard ratios (HRs) of 2.26, 1.84, 2.23, 1.51, and 2.06, respectively, whereas chemotherapy and radiotherapy could significantly reduce the death risk by 39% for patients with lung adenocarcinoma (*P* < 0.05) (Fig. 3 and Supplemental Fig. 1). In stratified analysis, enhanced IFT20 staining intensity score was significantly decreased the risk of death by 16% for patients without distant metastasis. And elevated the IOD/area of GM130 expression significantly decreased the death risk of lung adenocarcinoma patients with tumor size > 7 cm or distant metastasis by 54% and 65%, respectively (Table 4).

**Table 4:** Effects of IFT20 and GM130 protein expressions on the survival of patients with lung adenocarcinoma by stratified analysis

Proteins	Variables	Differentiated types		Tumor size		TNM stage		Lymphatic metastasis		Distant metastasis	
		Poorly	moderately to well	> 7 cm	≤7 cm	II ~ IV	I	Yes	No	Yes	No
IFT20	IOD/area	1.17(0.93,1.48)	0.89(0.74,1.07)	0.84(0.47,1.52)	1.00(0.85,1.18)	0.98(0.83,1.15)	1.02(0.67,1.56)	1.03(0.85,1.25)	0.92(0.74,1.14)	1.07(0.78,1.46)	0.97(0.82,1.16)
	Rate of positive cells	1.01(0.99,1.02)	1.00(0.99,1.01)	1.03(0.99,1.07)	1.00(0.99,1.01)	1.00(0.99,1.01)	0.99(0.96,1.02)	1.00(0.99,1.01)	1.00(0.98,1.01)	1.02(1.00,1.04)	1.00(0.99,1.01)
	Staining intensity score	1.12(0.87,1.45)	0.85(0.70,1.04)	0.91(0.53,1.56)	0.94(0.80,1.10)	0.87(0.74,1.03)	1.07(0.66,1.73)	0.93(0.76,1.12)	0.92(0.67,1.27)	1.37(0.97,1.94)	0.84(0.71,0.99)
GM130	IOD/area	0.86(0.59,1.27)	0.95(0.80,1.12)	0.46(0.26,0.82)	0.98(0.82,1.18)	0.91(0.76,1.08)	1.07(0.69,1.67)	1.01(0.80,1.27)	0.91(0.74,1.12)	0.35(0.20,0.62)	0.99(0.98,1.19)
	Rate of positive cells	1.00(0.98,1.02)	0.99(0.97,1.01)	0.96(0.92,0.99)	0.99(0.98,1.01)	0.99(0.98,1.01)	1.02(0.99,1.06)	1.00(0.99,1.02)	0.98(0.96,1.01)	0.97(0.95,0.99)	0.99(0.98,1.01)
	Staining intensity score	0.98(0.77,1.25)	0.89(0.75,1.06)	0.85(0.50,1.44)	0.93(0.80,1.07)	0.88(0.76,1.02)	1.33(0.87,2.01)	0.93(0.78,1.11)	0.88(0.69,1.14)	0.75(0.58,0.97)	0.92(0.78,1.08)

## Discussion

In this study, we found the proportions of some clinicopathological features including T3 ~ T4 stage, N2 ~ N3 stage and lymphatic metastasis were significantly lower in patients with IFT20 positive expression than those with negative expression. And the average OS of patients was much longer in IFT20 positive expression group. Both the proportion of poorly differentiated type cells and the tumor size were significantly less in GM130 positive expression group when compared with the negative expression group. Multivariate logistic regression analysis was further confirmed that increased IFT20 protein expression was significantly associated with TNM stage in II ~ IV and lymphatic metastasis, whereas there was a significantly negative relationship between GM130 protein expression and poorly differentiated type cells and tumors size> 7 cm. And Cox proportional hazard models exhibited that poorly differentiated type cells, tumor size > 7 cm, TNM stage II ~ IV, lymphatic and distant metastasis were the risk factors for the survival of patients with lung adenocarcinoma. Consistent with our findings, a large amount of evidence has revealed that patients with lung adenocarcinoma have a poor prognosis when the tumor was poorly differentiated, in advanced TNM stages and with distant metastasis [19–22]. The micropapillary and solid subtypes classified as poor differentiation seems to be the most adverse prognostic factor [19, 20]. The IFT20 and GM130 protein expressions were only negatively associated with the death risk of patients with specific clinicopathological feature such as tumor size > 7 cm or distant metastasis. These findings could indicate that IFT20 and GM130 protein expressions may indirectly affect survival time by influencing some clinicopathological features which are associated with the survival of patients with lung adenocarcinoma.

Primary cilium, a sensory appendage on the cell surface has a strong role in cell cycle control, differentiation and polarity that is correlated with prognosis of adenocarcinoma [23]. IFT20 is the smallest subunit of IFT complex, which is crucial for the formation of primary cilium within a cell [8]. Our results showed that the expression of IFT20 protein was significantly higher in cancerous tissues than that in matched adjacent tissues. A study reported that primary cilia frequency was significantly elevated in lung adenocarcinoma when compared with normal tissues [6], which may be partially caused by increased IFT20 expression in cancer cells. However, we found a significantly negative association of IFT20 staining intensity score with clinicopathological features like TNM stage and lymphatic metastasis of patients with lung adenocarcinoma. It is suggested that IFT20 might be a promoter of tumorigenesis, whereas it functions as a negative regulator in the progression of invasion and metastasis of lung adenocarcinoma cells. These seemingly paradoxical effects of IFT20 could be explained by a dual role of primary cilia mediated Hh signaling activation and suppression in oncogenesis and tumor progression [24]. Consistent with our results, Yang et al. reported that the level of IFT20 protein was negatively correlated with the malignancy of breast cancer cells, in which deletion of IFT20 promoted epithelial-mesenchymal transitions (EMT) and enhanced the migration of breast cancer cells by decreasing F-actin associated protein Transgelin-2 (TAGLN2) expression [25]. Overexpression of TAGLN2 could suppress lung metastasis in a mouse model of breast cancer [26]. A previous publication has identified that TAGLN2 was upregulated in patients with lung adenocarcinoma, most of whom were in the early stage of lung adenocarcinoma [27]. It is thus possible that negative relationship of IFT20 expression with TNM stage and lymphatic metastasis due to its role in regulating elevated synthesis of TAGLN2 in the early step of carcinogenesis, a hypothesis that will need to be explored in detail in the future.

A recent study has proposed that invasion of lung adenocarcinoma is associated with loss of cell polarization [28], which is known as a typical feature of EMT that promotes epithelial tumorigenesis and metastasis [29]. Some studies have indicated that the Golgi complex has been found to play a key role in promoting cell polarization [13, 14]. As a Golgi marker, GM130 protein is involved in the maintenance of Golgi structure and the process of intracellular proteins transport to regulate the orientation of cell migration and polarity [15, 16]. Depletion of GM130 resulted in a reduction of E-cadherin expression in epithelial cells, which is considered to be an indicator of loss in cell polarity and epithelial identity [15]. It has been confirmed that a reduction in membranous E-cadherin/catenin complex expression was strongly associated with poor differentiation, distant metastasis and post-operative recurrence of patient with lung adenocarcinoma [30, 31]. Therefore, the role of GM130 in regulating E-cadherin expression of epithelial cells may be used to explain our results of negative association between GM130 expression and the risk of poor differentiation, tumor size and shortened OS of patients with lung adenocarcinoma, which would be interesting to study further.

To the best of our knowledge, this is the first study to investigate the associations of IFT20 and GM130 protein expressions with clinicopathological features and survival of patients with lung adenocarcinoma. However, there are two limitations. Firstly, this is a case study in which the number of subjects is relatively small. But each patient’s matched adjacent tissue could be considered as a self-control, which can reduce the bias to some extent. Secondly, although both IFT20 and GM130 proteins are localized in the Golgi apparatus and interact with each other, their interaction and mechanism on the development of lung adenocarcinoma could not be evaluated. We only found that IFT20 protein was associated with TNM stage in II ~ IV and lymphatic metastasis, whereas GM130 protein was correlated with poorly differentiated type cells and tumors size > 7 cm. The influences of these two proteins interaction on clinicopathological features of lung adenocarcinoma need to be explored in animal experimental models or case-control studies in the future.

## Conclusions

In conclusion, we found the expressions of IFT20 and GM130 protein were negatively associated with clinicopathological features including tumor differentiated types, size, TNM stage and lymphatic metastasis of lung adenocarcinoma. IFT20 and GM130 proteins have some protective effects on the survival of lung adenocarcinoma patients with specific clinicopathological features such as large tumor size and distant metastasis. Thus, it is indicated that IFT20 and GM130 protein could play crucial roles in the development of lung adenocarcinoma.

## Supplementary Information

**Additional file 1 Supplemental Table 1.** The correlation between IFT20 and GM130 protein in cancerous and adjacent tissues. **Supplemental Fig. 1.** The forest plot of Cox proportional hazard regression models with TNM stage included. To exclude collinearity, the three types' expressions of IFT20 and GM130 protein (IOD/area, rate of positive cells, and staining intensity score) were separately included in the model.

## Data Availability

The data used to support findings of this study are available from the corresponding author upon request.

## Abbreviations

NSCLC: Non-small cell lung cancer
OS: Overall survival
IFT20: Intraflagellar transport protein 20
GM130: Cis-Golgi matrix protein 130
AKAP450: A-kinase anchor protein 450
TNM: Tumor node metastasis
HE: Hematoxylin and eosin
IHC: Immunohistochemistry
TBS: Tris-buffer solution
IOD/area: Mean optical density
OR: Odd ratio
CI: Confidence interval
HRs: Hazard ratios
EMT: Epithelial-mesenchymal transitions
TAGLN2: Transgelin-2
