# Supplementary material for: Associations of IFT20 and GM130 protein expressions with clinicopathological features and survival of patients with lung adenocarcinoma
Source: BMC Cancer. 2022 Jul 22;22:809. doi: 10.1186/s12885-022-09905-6 (PMC9308367; doi:10.1186/s12885-022-09905-6)
Supplement: Supplementary file 1 — Additional file 1 Supplemental Table 1. The correlation between IFT20 and GM130 protein in cancerous and adjacent tissues. Supplemental Fig. 1. The forest plot of Cox proportional hazard regression models with TNM stage included. To exclude collinearity, the three types' expressions of IFT20 and GM130 protein (IOD/area, rate of positive cells, and staining intensity score) were separately included in the model. [file 12885_2022_9905_MOESM1_ESM.doc]

Supplemental Table 1 The correlation between IFT20 and GM130 protein in cancerous and adjacent tissues

| Protein | IOD/Area of IFT20 | |  | IFT20 positive cells rate | |  | IFT20 staining intensity scores | |
| --- | --- | --- | --- | --- | --- | --- | --- | --- |
|  | C | A |  | C | A |  | C | A |
| GM130 |  |  |  |  |  |  |  |  |
| C | 0.223* |  |  | 0.032 |  |  | 0.492* |  |
| A |  | 0.385* |  |  | 0.040 |  |  | 0.424* |

C: Cancerous tissue; A: Adjacent tissue.


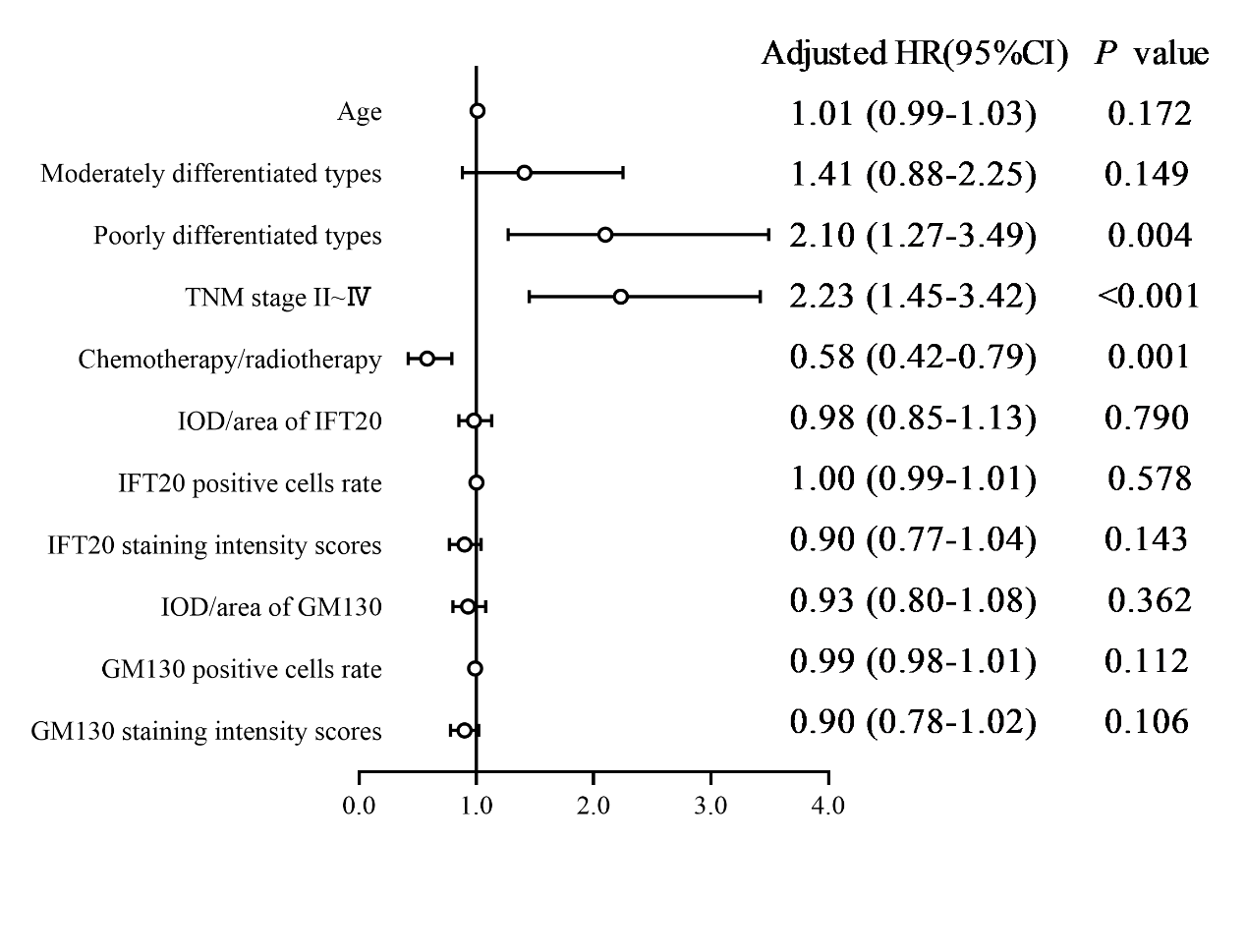


Supplemental Figure 1 The forest plot of Cox proportional hazard regression models with TNM stages included. To exclude collinearity, the three types’ expressions of IFT20 and GM130 protein ((IOD/area, rate of positive cells, and staining intensity score) were separately included in the model.
